# QG-WRN: A Quantum-Enhanced Graph Convolutional Wide Residual Network for ASD Diagnosis via Neuroimaging Sensing Technology

**DOI:** 10.3390/s26133997

**Published:** 2026-06-24

**Authors:** Nanting Huang, Xiaoyu Li, Xin Yang, Li Xie, Guowu Yang, Liujiang Zhou

**Affiliations:** 1 The School of Physics, University of Electronic Science and Technology of China, Chengdu 611731, China; nantingh@163.com; 2 The School of Information and Software Engineering, University of Electronic Science and Technology of China, Chengdu 611731, China; 18151061560@163.com; 3 The School of Computer Science and Engineering, University of Electronic Science and Technology of China, Chengdu 611731, China; xin.yang@std.uestc.edu.cn (X.Y.); guowu@uestc.edu.cn (G.Y.)

**Keywords:** autism spectrum disorder (ASD), quantum graph convolutional network (QGCN), dual-stream architecture

## Abstract

The pathological mechanism of autism spectrum disorder (ASD) exhibits dual heterogeneity: abnormal local energy metabolism and brain-wide high-order topological failure. To synergistically characterize these complex signals captured by advanced neuroimaging sensors, we propose the Quantum-Enhanced Graph Convolutional Wide Residual Network (QG-WRN), a modality-specific, decoupled parallel dual-stream architecture. In the classical branch, to accurately capture the spatial distribution of local metabolic abnormalities, we employ a wide residual network (WRN) to extract amplitude of low-frequency fluctuation (ALFF) features, leveraging its expanded feature channels to effectively mine regional neurodynamic properties. Furthermore, to overcome the representational bottlenecks of classical linear operators in parsing hidden, long-range network connections, we introduce quantum computing, exploiting its exponentially expansive state space and intrinsic low-parameter regularization mechanism. Guided by these properties, the quantum branch utilizes a variational quantum graph convolutional (QGCN) module—featuring a trainable circular encoding strategy and a hardware-efficient 4-qubit configuration—with a 2-layer nested message passing structure to process the functional connectivity (FC) matrix, harnessing quantum interference in Hilbert space to parse complex topology while effectively mitigating overfitting on small-sample medical data. A unified training scheme achieves full-dimensional fusion of node activity and topology. Achieving 68.49% accuracy, our method outperforms 10 classic and recent new baselines, providing a powerful computational intelligence tool for sensor-based ASD clinical diagnosis. Furthermore, interpretability analysis successfully maps core disease hubs to standard AAL116 atlas coordinates, providing a powerful tool for computationally aided ASD diagnosis.

## 1. Introduction

With the rapid advancement of neuroimaging sensing technology, resting-state functional magnetic resonance imaging (rs-fMRI) has become a pivotal data source for exploring the functional organizational patterns of the human brain. The high-dimensional spatiotemporal data generated by these advanced neuro-sensors harbor rich biomarker information, serving as critical inputs for intelligent sensing-aided diagnosis (SAD) systems. In the realm of neurodevelopmental disorders, specifically regarding the automated identification of autism spectrum disorder (ASD), extracting discriminative features from complex sensing data remains a focal point at the intersection of pattern recognition and computational neuroscience [1].

In contrast to task-fMRI, which measures localized brain activations evoked by specific cognitive stimuli, rs-fMRI operates as a continuous, passive neuro-sensor. It captures the spontaneous low-frequency fluctuations of the BOLD signal when the subject is at rest. This modality is particularly suited for ASD diagnosis, as ASD is a pervasive developmental disorder fundamentally characterized by alterations in intrinsic whole-brain network architectures (e.g., Default Mode Network) that are most prominently observable in the baseline resting state.

In the continuous exploration of automated diagnosis, the research community has established a solid foundation utilizing traditional machine learning, such as support vector machines (SVMs) [2]. As deep learning evolved, researchers sought to capture complex imaging features. For instance, some convolutional neural network (CNN) architectures integrated the local amplitude of low-frequency fluctuation (ALFF) and global functional connectivity (FC) [3]. To address multi-scale feature extraction, Khosla et al. [4] utilized 3D CNNs to capture the spatial heterogeneity of functional connectomes. Furthermore, Transformer architectures [5] have been introduced to characterize long-range dependencies using global self-attention mechanisms. While these architectures have significantly advanced the field, processing the dual heterogeneity of ASD brain networks—specifically the interplay between local metabolic signals and global topologies—presents a unique computational challenge. In “high-dimensional, small-sample” medical imaging scenarios, models with immense parameter spaces, such as Transformers, face the demanding task of maintaining generalization. Meanwhile, forcing the coupling of multi-scale features may inadvertently smooth out highly discriminative local spatial heterogeneity.

Recent vision-oriented and graph-oriented transformer models, including ViT, Swin Transformer, Brain Network Transformer, and Graphormer-style spatial encodings [6,7,8], further demonstrate the importance of non-local dependency modeling in high-dimensional visual and connectomic data. These studies motivate global relation modeling, while also highlighting the need for compact representations in high-dimensional, small-sample neuroimaging tasks.

To more naturally characterize topological structures, graph representation learning has been increasingly applied. Wang et al. [9] validated the efficacy of multi-atlas graph convolutional networks focusing on node attributes, while Chen et al. [10] integrated multimodal deep learning with clinical phenotypes. Furthermore, recent advancements have demonstrated the remarkable efficacy of graph convolutional aggregation models in leveraging skip connections and identity mapping to accurately classify neurodevelopmental and brain disorders [11]. However, linear message-passing mechanisms in classical graph neural networks typically exhibit a low-pass filtering tendency during whole-brain multi-layer aggregation [12]. This characteristic can inadvertently dilute the subtle, high-order long-range topologies that are highly informative for understanding cognitive impairments in ASD [13]. Furthermore, balancing logical interpretability [14] with high generalization robustness across multi-site data [15] remains an ongoing area of refinement. Subsequent studies have explored improved graph attention networks (GATs) [16,17], adversarial graph neural networks [18], and multi-scale dynamic graph learning (MDGL) [19] to enhance feature representation.

Fundamentally, the core challenge lies not in the design of previous models, but in the inherent computational difficulty of processing high-dimensional, strongly nonlinear correlations with standard linear operators [20]. This observation suggests that introducing mechanisms with greater dimensional elasticity, such as quantum machine learning (QML) [21], could provide a valuable refinement to existing computational paradigms. By leveraging the unique properties of quantum mechanics, we can offer a new perspective on synergistically optimizing the fusion of local regional space and global network topology.

Inspired by recent advancements in hybrid quantum-classical computing [22,23], this paper proposes the modality-specific decoupled Quantum-Enhanced Graph Convolutional Wide Residual Network (QG-WRN). Rather than directly concatenating local node activity with large-scale topological networks, we seek to refine the feature integration process through a dual-stream parallel processing architecture. In the classical computing branch, a feature reordering strategy combined with a wide residual network (WRN) is introduced for the amplitude of low-frequency fluctuation (ALFF) data, utilizing a multi-receptive field mechanism to focus on spatial feature distributions and improve the extraction efficiency of local metabolic signals. Concurrently, in the quantum computing branch, the FC matrix is processed through a dedicated variational QGCN module [24]. By mapping the complex, non-Euclidean high-order topological features into the exponentially dimensional Hilbert space, the model adaptively captures deep pathological associations. Notably, benefiting from the endogenous regularization effect of trainable quantum parameters, the model achieves higher parameter efficiency, thereby effectively mitigating the overfitting risk in small-sample medical environments.

The main contributions of this study are summarized as follows:

1. Proposing the modality-specific decoupled dual-stream QG-WRN architecture. By refining the traditional feature coupling paradigm and utilizing a WRN with feature reordering to extract local metabolic features, it achieves a deep synergistic fusion of local activity and global topology.
2. Introducing a variational QGCN module for global feature extraction. Configured with a 2-layer nested message passing structure, it captures high-order nonlinear topologies through quantum state evolution in a high-dimensional space, effectively alleviating the overfitting problem of medical small-sample data with higher parameter efficiency. Furthermore, the integration of trainable parameters during quantum state evolution enhances the discriminability of ASD pathological features.
3. Demonstrating efficacy through extensive comparative and ablation experiments. The experimental results verify that the proposed method provides a novel and reliable refinement for the challenges of high-order topology extraction and synergy in brain networks.

## 2. Materials and Methods

### 2.1. Problem Motivation and Definition

The pathological analysis of ASD in computational neuroscience is increasingly focused on the synergistic manifestation of local metabolic heterogeneity and global topological imbalances. From a clinical perspective, localized abnormalities in the ALFF reflect regional neurodynamic disturbances, while FC patterns characterize brain-wide communication efficiency. However, effectively integrating these two distinct yet complementary modalities remains a non-trivial challenge.

Traditional diagnostic frameworks often rely on brute-force feature concatenation, where ALFF and FC are flattened into a single vector for processing by linear classifiers like SVM or standard deep models such as 1D-CNN. We observe that this paradigm may inadvertently disrupt the intrinsic spatial manifold of high-order pathological features. The limited exploration of decoupled dual-stream architectures in previous works primarily stems from two factors:

1. High-level models with extensive receptive fields, such as Transformers, typically require massive datasets to optimize their immense parameter space, making them prone to overfitting in the “high-dimensional, small-sample” scenarios characteristic of medical imaging;
2. Conventional graph neural networks, while designed for topology, inherently suffer from over-smoothing and information bottleneck effects when processing dense whole-brain networks. Mathematically, standard message-passing mechanisms act as a form of Laplacian smoothing. Let **A** be the functional connectivity adjacency matrix and $\tilde{A}=D^{-1/2}(A+I)D^{-1/2}$ be its symmetrically normalized counterpart. The linear feature aggregation at layer l is strictly defined as $H^{\left(l+1\right)}=\sigma (\tilde{A}H^{\left(l\right)}W^{\left(l\right)})$.

As the network depth increases (l→∞), the node representations $H^{\left(l\right)}$ mathematically converge towards a stationary subspace spanned by the principal eigenvector of $\tilde{A}$. In the context of highly connected brain functional networks, this inherent low-pass filtering tendency exponentially dilutes high-frequency spatial signals, effectively erasing the subtle, long-range high-order connections that are crucial for capturing ASD cognitive impairments. Furthermore, compressing these complex, non-Euclidean anatomical topologies into a fixed-dimensional Euclidean space ${\mathbb{R}}^{d}$ inevitably triggers an information bottleneck (often termed “over-squashing”). Here, critical structural dependencies are irreversibly lost due to the limited dimensional elasticity of classical continuous spaces.

To refine these limitations, we define the ASD diagnostic task as a modality-specific feature extraction problem. Specifically, we utilize a WRN to focus on the spatial distribution of regional metabolism, leveraging its expanded width to mine nonlinear patterns in ALFF without necessitating extreme depth. For global topology, we introduce quantum state evolution as a refinement to classical linear operators. By mapping the FC matrix into an exponentially expansive Hilbert space, the variational quantum circuits (VQC) can capture hidden, linearly inseparable high-order associations through quantum interference and entanglement. This approach achieves higher parameter efficiency, providing a robust physical regularization that mitigates the risk of memorizing small-sample noise. As verified in our subsequent ablation studies, this decoupled dual-stream design is essential for preserving the distinct pathological signals of each modality.

### 2.2. Model Architecture of QG-WRN

To synergistically characterize the “local metabolic heterogeneity” and “global topological imbalance” present in the brain functional networks of ASD patients, this study proposes a modality-specific decoupled dual-stream hybrid architecture: the QG-WRN. The proposed QG-WRN architecture implements a parallel sensing-information processing paradigm, decoupling the raw neuro-sensing data into independent streams to preserve the distinct manifold of each modality. Through a unified training scheme, this approach aims to achieve an accurate representation of complex neuropathological features.

**Figure 1:**
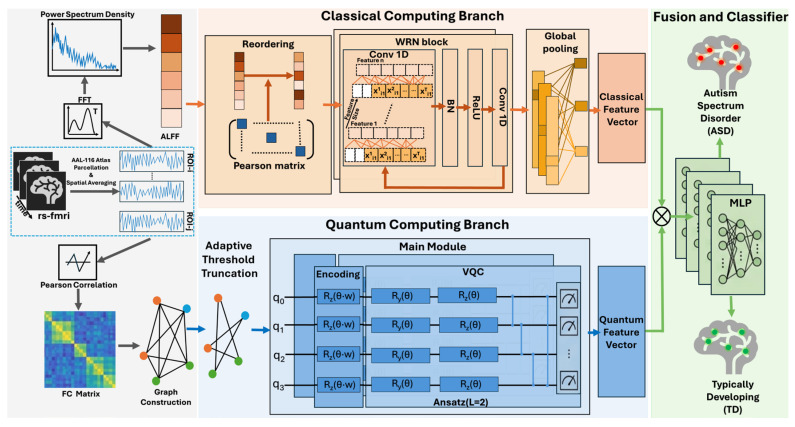
Overview of the QG-WRN.

As illustrated in Figure 1, the QG-WRN operates through a decoupled, parallel architecture comprising two complementary functional branches. The classical computing branch specifically models the local amplitude of low-frequency fluctuation features extracted from rs-fMRI neuro-sensors. To optimize spatial representation, we first introduce a feature reordering strategy, followed by the implementation of a WRN as the backbone module. Utilizing the spatial feature mining capabilities of its widened receptive fields, this branch aims to capture abnormal local energy metabolism signals triggered by pathological changes in specific anatomical brain regions. Concurrently, in the quantum graph convolutional branch, the functional connectivity matrix—which reflects whole-brain scale sensing interactions—is independently isolated and fed into the variational QGCN module. Configured with a 2-layer nested message passing structure, this module leverages the evolution and interference of qubits within a high-dimensional Hilbert space to adaptively parse complex, high-order nonlinear connection patterns that are frequently overlooked by classical operators. Furthermore, the inherent low-parameter physical regularization of the quantum circuits significantly improves the model’s generalization robustness in small-sample clinical environments.

In the preprocessing stage (within the gray box in Figure 1), the extraction of temporal signals from spatial rs-fMRI volumes involves parcellating the brain into 116 Regions of Interest (ROIs) using the AAL atlas, followed by averaging the voxel-wise BOLD signals within each ROI to generate 1D time-series.

#### 2.2.1. WRN Model

**Figure 2:**
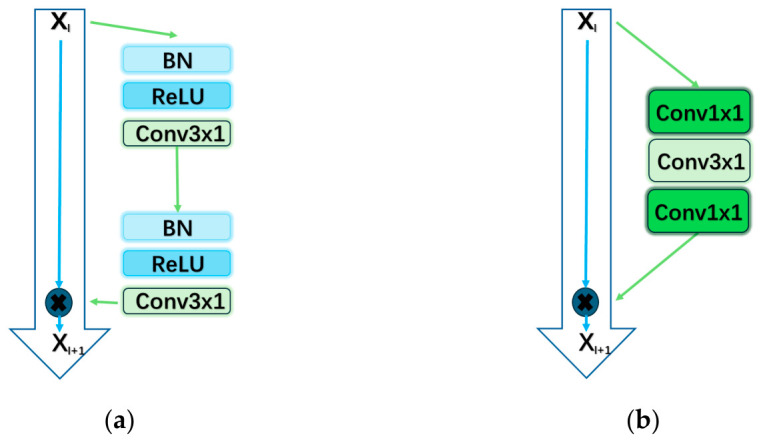
Structure of WRN model. (**a**) Basic convolutional layer; (**b**) Bottleneck layer.

WRN, proposed by Zagoruyko et al. [25], is an advanced variant of ResNets designed to improve performance by increasing layer width rather than depth. The WRN branch in this study prioritizes the widening factor over extreme network depth, where this factor is implemented by expanding the number of feature channels within the residual blocks. Specifically, expanding the feature maps from 32 to 64 enables the simultaneous extraction of multiple low-frequency oscillation patterns, which facilitates a more robust characterization of regional neurodynamic properties. Figure 2 illustrates the structure of the WRN model. This architecture is employed to mine nonlinear pathological features within the ALFF of 116 brain regions, thereby facilitating the acquisition of more comprehensive feature representations when processing one-dimensional neuroimaging vectors.

The fundamental unit of WRN is the wide residual block. Let $x_{l}$ and $x_{l+1}$ denote the input and output of the l-th residual unit, respectively; their mapping relationship can be expressed as:

(1) $$x_{l+1}=x_{l}+F(x_{l},W_{l})$$

where F represents the residual function, and $W_{l}$ denotes the parameters of the l-th residual unit. In the adopted WRN architecture, a pre-activation mechanism is utilized, wherein normalization and activation precede the convolution operations. For the l-th residual block, the computational flow of its internal function F is defined as follows:

(2) $$z_{l}=\sigma (\mathrm{BN}(x_{l}))$$

(3) $$F(x_{l})={\mathrm{Conv}}_{3\times 1}^{\left(2\right)}\left(\sigma \left(\mathrm{BN}\left({\mathrm{Conv}}_{3\times 1}^{\left(1\right)}(z_{l})\right)\right)\right)$$

where BN denotes batch normalization, and σ represents the ReLU activation function. Each convolutional layer employs a 1D convolutional kernel of size 3 (Conv 3 × 1), with $L_{2}$ regularization applied to mitigate overfitting. To manage variations in feature dimensions (filters) or reductions in spatial stride, bottleneck blocks are introduced. These blocks consist of a 3 × 1 conv layer surrounded by 1 × 1 conv layers, which first reduce and subsequently restore dimensionality. Specifically, when the channel dimension of the input $x_{l}$ mismatches the output of the residual path, the shortcut connection undergoes a linear transformation via a 1 × 1 convolutional layer $W_{s}$:

(4) $$x_{l+1}=F(x_{l},W_{l})+W_{s}x_{l}$$

During the feature extraction phase, the network initially processes the input through a 32-channel initial convolutional layer, followed sequentially by two residual stages (with feature dimensions of 32 and 64, respectively). To transform the multi-dimensional feature maps into fixed-length regional feature representations, global average pooling is applied at the output end. Ultimately, a fully connected layer with a tanh activation function maps the local features into a 2-dimensional compact vector.

Simultaneously, to address the high-dimensional redundancy inherent in medical data, a feature reordering strategy is implemented in this branch. Traditional CNNs rely on the spatial locality of input data. However, under the standard AAL116 atlas anatomical ordering of ALFF features, discriminative brain regions (e.g., the posterior cingulate cortex and prefrontal cortex) are often discretely distributed within the vector space. This spatial separation makes it difficult for classical convolutional kernels to simultaneously capture the synergistic variations in these key regions within a limited receptive field. Following the reordering process, the model performs feature mapping on the original matrix and conducts spatial aggregation with the thresholded adjacency matrix. By clustering high-contribution brain regions within the tensor space, a logical spatial proximity is effectively constructed.

The spatial reordering step changes only the one-dimensional tensor neighborhood seen by the WRN kernels; it does not alter biological ROI identity or the FC graph. By placing functionally similar ROIs closer in the tensor, local convolutional filters can better capture coordinated neurodynamic perturbations among regions that are distant under the default atlas order.

For graph reconstruction, each node corresponds to one AAL116 ROI and carries explicit regional information: the local ALFF intensity used by the classical branch and the FC-derived topological descriptor used before quantum preprocessing. Edges are weighted by Pearson correlation coefficients between mean ROI time series. The adjacency matrix is symmetrized, weak edges are removed by the |**r**| ≥ 0.2 cutoff, and the remaining weighted topology is normalized before graph-level pooling.

#### 2.2.2. QGCN Model

For the specific task of processing whole-brain FC matrices in ASD identification, traditional graph-based methods encounter certain representational limitations. In brain network modeling, standard neighborhood message-passing mechanisms may lead to the smoothing of regional features during multi-layer aggregation, which can make it challenging to preserve the subtle, long-range connection signals that are often characteristic of ASD-related topological changes. Furthermore, when dealing with the high-dimensional, small-sample data typical of neuroimaging, increasing the depth of classical architectures to capture nonlinear associations often introduces a notable risk of overfitting.

To refine the characterization of complex topological features in this diagnostic task, this branch processes the global FC matrix using a variational QGCN module. This approach integrates the linear aggregation of graph structures with the nonlinear evolution of quantum circuits, aiming to provide a more nuanced representation space for high-order brain network associations.

To reduce the impact of potential physiological noise on the evolution of quantum states, this paper adopts an adaptive threshold truncation mechanism for the input functional connectivity adjacency matrix **A**, as shown in Formula (5). Combining mature network neuroscience theories and the latest achievements in graph machine learning [26,27,28,29], this paper selects 0.2 as an empirical baseline threshold. This truncation operation enhances the sparsity of the graph, effectively filtering out weak, potentially spurious correlations driven by physiological noise and providing a stable topological structure with an improved signal-to-noise ratio prior to quantum state evolution. Recent studies on brain connectivity groups have shown that setting a reasonable threshold can eliminate false positive edges generated by physiological noise, thereby preserving the true community structure and clustering features of the brain network. In addition, from an algorithmic perspective, multiple benchmark studies on graph neural network-based brain connectivity omics have shown that high-density maps without threshold processing will inevitably suffer from serious over smoothing problems during neighborhood aggregation. By removing weak and false correlations, this method can effectively avoid the problem of excessive smoothing caused by high-density maps and obtain an initial topology structure with excellent signal-to-noise ratio. This is crucial for accurately extracting high-order pathological invariant features using quantum phase evolution in the future.

(5) $$A_{ij}^{\prime }=A_{ij}\cdot I(|A_{ij}|>0.2)$$

where I(•) represents the indicator function. The aggregated node features are compressed into a graph-level feature vector via global average pooling, and then processed through batch normalization and a hyperbolic tangent activation function, ultimately yielding an 8-dimensional quantum preprocessing feature vector $x\in {\mathbb{R}}^{d}$. This preliminary step effectively filters out weak noise connections with small absolute values, providing an initial input with a high signal-to-noise ratio for the subsequent quantum state evolution.

After generating the preprocessing vector x, the information is injected into a variational quantum circuit comprising N qubits (N = 4 in this study). Because the input dimension d exceeds the number of qubits, a parameterized data re-weighting circular encoding strategy is adopted to prevent information loss, effectively encoding the entire 8-dimensional classical feature set into 4 qubits. Additionally, to address the limitation that static encoding in traditional quantum graph models adapts poorly to complex manifolds, this study incorporates the trainable quantum state encoding mechanism proposed by Ovalle-Magallanes et al. [30]. Diverging from traditional fixed-angle encoding, the model introduces trainable weight parameters into the angles of the quantum rotation gates.

The circular encoding addresses the apparent 8-to-4 dimensional discrepancy by distributing the 8 preprocessing features across two encoding cycles. Feature $x_{j}$ is assigned to qubit q=j mod 4, while the cycle index determines the trainable rotation scale applied to that feature. The same qubit can therefore receive multiple feature-dependent rotations, storing compressed information in amplitudes and relative phases rather than discarding half of the input vector.

**Figure 3:**
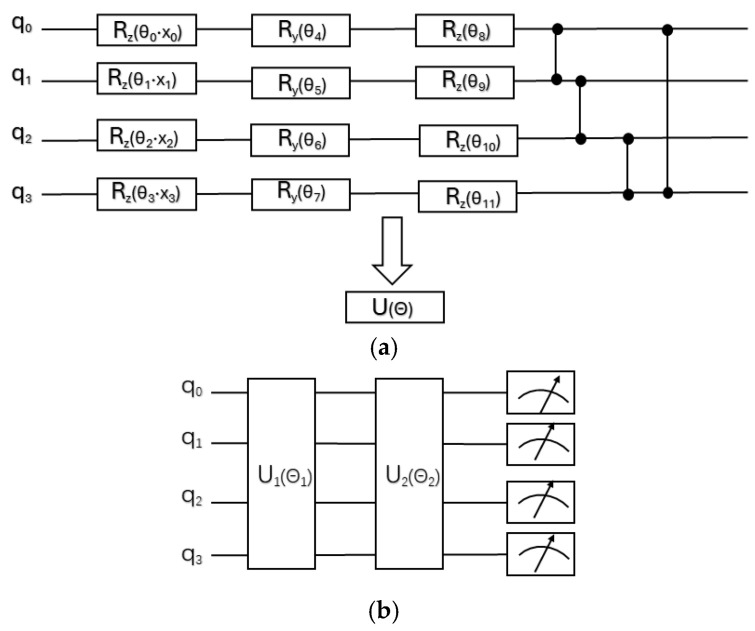
The variational quantum circuits. (**a**) Explicit gate-level diagram of quantum neural network circuit; (**b**) Complete quantum circuit.

Within the quantum circuit illustrated in Figure 3, during each evolution layer $U\left(\Theta \right)$, the model employs three cascaded rotation gates for the i-th qubit to execute feature extraction:

(6) $$U_{rot}^{\left(q\right)}(\theta ,x)=R_{z}(\theta_{k+2})R_{y}(\theta_{k+1})R_{z}(\theta_{k}\cdot x_{idx})$$

where idx=(L•N+q)mod d represents the classical feature index corresponding to the current qubit. In this encoding mechanism $R_{z}(\theta_{k}\cdot x_{idx})$, the classical feature $x_{idx}$ is not directly input as a fixed phase angle. Instead, it initially undergoes dynamic coupling with a trainable physical parameter $\theta_{k}$. Through backpropagation optimization, the model can adaptively adjust $\theta_{k}$, thereby effectively amplifying the phase difference $\Delta \phi =\theta_{k}\cdot \Delta x$ within the quantum space. This mechanism enables the model to accurately capture weak pathological signals concealed within strong background noise, addressing the limitation of classical operators in extracting hidden nonlinear connections.

To quantify the contribution of this mapping to the classification boundary, we introduce quantum state fidelity F to measure the similarity between two encoded quantum states $|\psi_{tc}\rangle$ and $|\psi_{\mathrm{asd}}\rangle$. Under idealized conditions, their cosine mapping relationship can be expressed as:

(7) $$F(|\psi_{\mathrm{tc}}\rangle ,|\psi_{\mathrm{asd}}\rangle )=|\langle \psi_{\mathrm{tc}}|\psi_{\mathrm{asd}}\rangle {|}^{2}\approx \cos^{2}\left(\frac{\theta \cdot \Delta x}{2}\right)$$

During the unified training process, the guidance of the loss function drives θ to seek the optimal observation angle, prompting the quantum state fidelity to approach 0. This implies that even if the original data heavily overlap in a low-dimensional space, the QGCN can drive them toward orthogonality within the high-dimensional Hilbert space, thereby effectively disentangling the feature manifolds at a physical level.

Subsequently, to capture high-order synergistic relationships across different topological dimensions, we introduce a strongly entangling structure composed of controlled-Z (CZ) gates acting on adjacent qubits:

(8) $$U_{ent}=\prod_{q=0}^{N-1}CZ(q,(q+1)\;\mathrm{mod}\;N)$$

When these graph topological features, having undergone adaptive distance maximization, enter the entangled state, the complex pathological connections that were previously intertwined in the low-dimensional classical space become effectively disentangled. The synergy of this dynamic mapping and high-dimensional entanglement overcomes the representational limitations inherent in classical GNNs.

After the 4 qubits undergo evolution through the L=2 layers of quantum gates, the final quantum state $|\psi_{out}\rangle$ encapsulates deep, whole-brain high-order topological information. By measuring the expectation value of each qubit in the Pauli-Z basis, the state is collapsed back into the classical space to form the final quantum feature vector $f_{q}(x)$:

(9) $$f_{q}(x)=\langle \psi_{\mathrm{out}}|Z_{q}|\psi_{\mathrm{out}}\rangle ,\;\;\;\;q\in \{0,1,\ldots ,N-1\}$$

This architecture demonstrates the structured dimensionality reduction and compact parameterization inherent to quantum computing. Although this quantum layer executes more complex nonlinear mappings in the Hilbert space compared to very deep traditional neural networks, the trainable parameters of the entire QGCN module depend solely on θ and are relatively few in number. This extreme parameter compactness establishes a robust intrinsic regularization mechanism. It enables the model to accurately separate hidden weak pathological connections of ASD on high-dimensional manifolds and mitigates the risk of the model memorizing small-sample noise through excessive parameterization, thereby improving robustness and generalization capabilities in medical small-sample scenarios.

#### 2.2.3. Feature Fusion and Classification

Following the parallel processing pipelines of the WRN and QGCN branches, the model extracts two distinct representations from the neuro-sensing data: a spatial feature vector capturing localized neurodynamic activity, and a quantum state feature vector encapsulating high-order topological associations within the brain network. To preserve the distinct manifold of each modality without early feature dilution, a concatenation operator is subsequently employed to fuse these modality-specific representations into a joint high-dimensional feature vector. This integrated vector is then fed into the classification head of a multi-layer perceptron (MLP) structure. To enhance the model’s generalization robustness and further explore the complex nonlinear interactions between the fused classical and quantum sensing features, the classifier incorporates fully connected layers strictly regularized by Dropout mechanisms. Finally, the joint representation is mapped to a binary diagnostic probability space via a softmax operator. This classification head directly anchors the loss function, thereby facilitating the end-to-end optimization of the entire single-stage architecture.

#### 2.2.4. Model Training Strategy

The binary cross-entropy loss function is adopted as the optimization objective of the model. The loss calculation formula is as follows:

(10) $$L=-\frac{1}{M}\sum_{m=1}^{M}[y_{m}\log({\hat{y}}_{m})+(1-y_{m})\log(1-{\hat{y}}_{m})]$$

where M represents the number of training samples. Through the Back-propagation algorithm, gradients flow simultaneously to the convolutional kernels of the classical WRN and the trainable rotary gate parameters $\theta_{k}$ of the VQC. This end-to-end collaborative training mechanism ensures that the feature reordering strategy and quantum nonlinear transformation evolve together towards maximizing classification accuracy, thereby fundamentally enhancing the model’s discriminative ability for complex neuroimaging data.

Gradient propagation through the hybrid model follows the chain rule over the classical-quantum interface. The classical feature x determines a rotation angle θ=αx+β. For a measured expectation value E(θ), the derivative with respect to theta can be evaluated by the parameter-shift rule, dE/dθ=[E(θ+π/2)−E(θ−π/2)]/2. This derivative is then multiplied by dθ/dx and propagated back to the preprocessing layer and WRN convolutional filters.

## 3. Experiments and Results

### 3.1. Data Pre-Processing

To facilitate the reliability of hybrid quantum-classical feature extraction, the rs-fMRI data for this study were sourced from the Preprocessed Connectomes Project (PCP) repository within the ABIDE I dataset, specifically utilizing the version processed via the CPAC pipeline. This pipeline incorporates standard neuroimaging processing stages, including despiking via 3dDespike, slice timing correction, motion correction, and registration of functional images to the MNI152 standard space. To further suppress physiological noise, the preprocessing involved the regression of 24 motion parameters, as well as white matter (WM) and cerebrospinal fluid (CSF) signals, followed by the application of a 0.01–0.1 Hz band-pass filter. Regarding potential artifacts within the PCP dataset, we implemented a rigorous quality control protocol by excluding subjects with a mean framewise displacement (Mean FD) exceeding 0.5 mm. This step was taken to support the integrity of the subsequent quantum neural network feature extraction. Consequently, a final cohort of 775 subjects (352 ASD and 423 TD) was retained for the analysis.

Based on the AAL-116 atlas, we extracted two categories of key features from the preprocessed derivative data. First, the ALFF was calculated from the signal time series to serve as an indicator reflecting the intensity of local spontaneous brain activity. Second, the whole-brain FC matrix was constructed by calculating the Pearson correlation coefficients between the average time series of the 116 ROIs. This dual-stream input mode, which integrates local neurodynamic features (ALFF) with global topological structures (FC), allows the QG-WRN architecture to characterize high-dimensional nonlinear pathological features of ASD within brain networks. This integrated representation effectively supports robust classification performance and enhances model interpretability.

To rigorously evaluate the generalization capability of the model and strictly preclude any potential data leakage, a stringent data partitioning and isolation protocol was implemented. The preprocessed samples were randomly partitioned into an 8:2 split. Specifically, 80% of the samples (620 subjects) formed the training set to optimize the WRN convolutional kernel weights and the trainable quantum parameters of the VQC, while the remaining 20% (155 subjects) served as an independent test set. Throughout this process, we meticulously preserved the original class distribution across both sets to avoid sample imbalance biases. Crucially, all data-driven preprocessing transformations—specifically the hierarchical clustering utilized for ALFF feature reordering—were computed exclusively on the training set. The derived spatial mapping indices were subsequently frozen and directly applied to the unseen test set. This rigorous isolation ensures that the test data remains entirely unexposed during both the feature engineering and the single-stage integrated training phases, thereby providing a highly robust and objective assessment of the parallel dual-stream architecture’s true diagnostic performance on clinical small-sample data.

### 3.2. Experimental Setup

#### 3.2.1. Software Environment

The public ABIDE I dataset comprises rs-fMRI data from typical control (TC) subjects and ASD patients across multiple sites. To enhance the extraction of spatial correlations by the 1D convolution, hierarchical clustering is utilized to reorder the indices of the 116 brain regions prior to WRN input. The hierarchical clustering matrix of feature rearrangement is only calculated on the current folded training set. Subsequently, the permutation index generated by the clustering is solidified and directly applied to the testing set. This operation positions functionally similar brain regions contiguously within the vector space, thereby providing a structured input that facilitates the convolutional kernels in capturing local synergistic patterns.

The model is implemented utilizing the TensorFlow 2.15 and Qiskit 0.25.0 frameworks. The Adam optimizer is employed in conjunction with a cosine annealing learning rate decay strategy. Specifically, the initial learning rate is set to 3 × 10^−4^ and gradually decays to 3 × 10^−5^ over 100 epochs, facilitating fine-grained convergence during the later stages of training. Concurrently, to address the challenge of sample imbalance, class weights are dynamically computed based on category frequencies during the training process (penalty weights: {TD: 0.9, ASD: 1.0}). Furthermore, to alleviate potential overfitting phenomena during model training, an early stopping method is adopted as a regularization strategy. This approach continuously monitors the training loss on an independent validation set. If the model fails to exhibit a noticeable reduction in validation loss over consecutive training epochs, a stopping mechanism is triggered. This strategy mitigates the risk of overfitting to the training data and supports the generalization capability of the model on unseen data.

#### 3.2.2. Quantum Circuit Configuration

To further ensure the feasibility of the QGCN module on current Noisy Intermediate-Scale Quantum (NISQ) hardware, we strictly control the complexity of the variational quantum circuit. For the 4-qubit architecture with L = 2 evolution layers, the implementation involves a total of 24 single-qubit rotation gates (R_y_ and R_z_) and 8 two-qubit CZ gates. The total circuit depth is kept relatively low to mitigate the accumulation of gate errors and decoherence effects inherent in current quantum processors. This lean configuration prioritizes higher parameter efficiency while maintaining sufficient expressive power to capture the non-linear topological features of brain networks, aligning with the practical constraints of near-term quantum devices.

#### 3.2.3. Statistical Metrics

To comprehensively evaluate the discriminative capability of the proposed QG-WRN model in the ASD identification task, four standard statistical metrics widely recognized in computational neuroimaging are employed: accuracy (ACC), sensitivity (SEN), specificity (SPE), and F1-score. Let TP, TN, FP, and FN denote the number of true positives, true negatives, false positives, and false negatives, respectively. These metrics are mathematically defined as follows:

(11) $$ACC=\frac{TP+TN}{TP+TN+FP+FN}$$

(12) $$SEN=\frac{TP}{TP+FN}$$

(13) $$SPE=\frac{TN}{TN+FP}$$

(14) $$F1=2\times \frac{Precision\times SEN}{Precision+SEN},\;Precision=\frac{TP}{TP+FP}$$

ACC serves as the fundamental global indicator of overall classification performance. SEN (also known as recall) and SPE assess the model’s reliability from two complementary clinical perspectives: the capability to correctly capture ASD pathological features and the ability to accurately identify typical development (TD) samples, respectively. Furthermore, to balance these two potentially conflicting dimensions, the F1-score—the harmonic mean of precision and sensitivity—is utilized. This metric provides a comprehensive measure of the model’s predictive effectiveness, serving as a critical indicator for evaluating the robustness of classification models when processing high-dimensional clinical sensing data.

### 3.3. Classification Comparison with SOTA Methods

To systematically evaluate the practical efficacy of the proposed architecture in computer-aided ASD diagnosis, a rigorous comparative study was conducted between the QG-WRN and 10 state-of-the-art baseline models. These comparison methods encompass classical machine learning algorithms, standard deep learning architectures, and GNN models.

As presented in Table 1, QG-WRN demonstrates competitive discriminative performance across multiple metrics, achieving a mean classification accuracy of 68.49% and a specificity of 74.29% on the test set. These results provide empirical evidence for addressing the computational challenges associated with the dual heterogeneity of brain networks. Compared to earlier models such as SVM (62.58%) and 1D-CNN (56.17%), the performance gain of the proposed method is notable. This performance gap stems from the limitation of classical shallow models and standard convolutional networks, which often forcibly couple ALFF and FC. Such simple concatenation tends to disrupt the spatial manifold of high-order pathological features, causing critical local abnormalities to be diluted by the surrounding data.

Further analysis of the graph learning paradigm shows that BrainGNN, specifically designed for medical brain networks, achieved an accuracy of approximately 60.65%. Although this model attempts to capture topological associations, it is constrained by the linear message-passing mechanism of classical GNNs, which inevitably induces a low-pass filtering effect during multi-layer neighborhood aggregation. This results in the over-smoothing of subtle nonlinear long-range pathological connections in ASD patients. In contrast, by introducing a quantum state evolution mechanism within a high-dimensional Hilbert space, QG-WRN captures high-order nonlinear connection patterns that are frequently missed by classical linear operators. Furthermore, in the high-dimensional, small-sample scenarios characteristic of medical imaging, deep models with massive parameter spaces, such as Video MAE and DRBMs, do not exhibit the expected generalization advantage. This further validates the effective balance between parameter constraints and feature representation achieved by the proposed model.

In summary, the superior diagnostic performance of the proposed QG-WRN over existing literature stems primarily from its architectural paradigm shift. Rather than employing the standard brute-force concatenation of multimodal features—which often leads to the dilution of critical local abnormalities—our modality-specific decoupled design independently preserves the spatial manifold of ALFF and the high-order topology of FC. Furthermore, compared to traditional GNN architectures that suffer from low-pass filtering, the introduction of the Hilbert space mapping via the compact 24-parameter VQC successfully amplifies weak, non-linear long-range pathological signals while inherently resisting the overfitting traps common in small-sample medical datasets.

**Figure 4:**
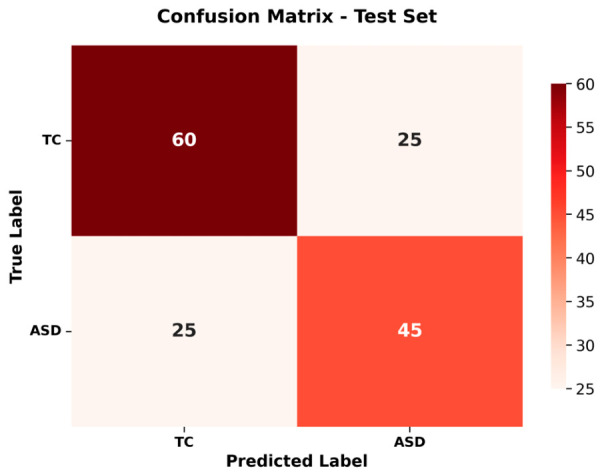
Confusion matrix of the proposed architecture evaluated on the independent test set.

To further exemplify the clinical usefulness and diagnostic reliability of the proposed architecture, we present the confusion matrix evaluated on the independent test set (Figure 4).

As illustrated, out of 155 test subjects, the model successfully identified 45 ASD patients and 60 Typical Control (TC) subjects. Notably, the model exhibits a highly balanced error distribution, with exactly 25 false negatives (ASD misclassified as TC) and 25 false positives (TC misclassified as ASD). This symmetrical distribution confirms that the classifier does not suffer from class imbalance bias and maintains a stable equilibrium between diagnostic Sensitivity and Specificity. From a clinical perspective, minimizing both the risk of delayed interventions (caused by missed diagnoses) and unnecessary psychological burden on healthy families (caused by false alarms) is paramount. The balanced diagnostic capability visualized in this confusion matrix validates the practical utility of the proposed method as a reliable computer-aided screening tool for ASD.

### 3.4. Ablation Study

To further dissect the specific contributions of each core component of QG-WRN to the overall discriminative performance, this study conducted detailed ablation experiments. By gradually stripping away key modules from the model, we observed changes in the network’s representational ability under the same set of validation criteria. The experimental results are presented in Table 2.

The necessity of the dual-stream decoupled architecture is validated through branch-removal experiments. When the model is stripped of the QGCN module (w/o QGCN) or the WRN module (w/o WRN), the overall accuracy exhibits a substantial decrease to 52.90% and a moderate reduction to 61.42%, respectively. These empirical data indicate that single-dimensional feature extraction is insufficient to characterize the complex pathological manifestations of ASD, whereas the designed modality-specific decoupling mechanism facilitates the full-dimensional synergistic integration of node activity and connection topology.

Simultaneously, the removal of feature reordering (w/o Reordering) and trainable quantum parameters (w/o Trainable Parameters) results in notable performance degradation. Significantly, this performance drop demonstrates that the diagnostic gains of the reordering strategy are rooted in the purposeful alignment of functional network modularity within the 1D spatial domain, rather than a simple disruption of the arbitrary default AAL116 atlas order. Hierarchical clustering strategically positions functionally correlated, yet anatomically distant, regions of interest contiguously within the tensor space. This targeted structural organization empowers the localized 1D convolutional kernels of the WRN to effectively harvest coordinated neurodynamic perturbations among synergistic brain regions, thereby suppressing high-dimensional redundancy and structurally maximizing inter-class distance.

Notably, when the quantum layer is degraded and replaced with a classical GNN (w/o Quantum Layer), the accuracy of the model regresses to 61.16%. To investigate the underlying physical mechanisms driving this phenomenon, this study conducts a comparative analysis of the parameter scales and dynamic training curves between the classical GNN and the QGCN under the unified training scheme.

**Figure 5:**
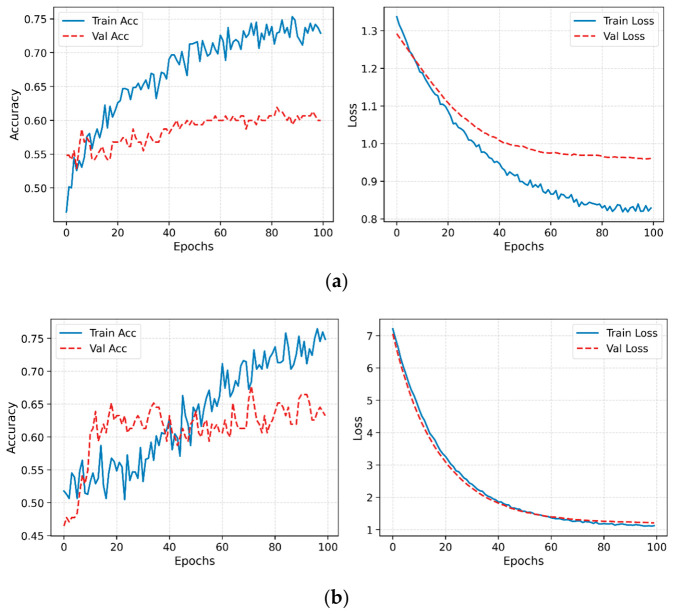
Training curve. (**a**) WRN + GNN; (**b**) QG-WRN.

As indicated by the statistics in Table 3, under equivalent topology extraction tasks excluding the fully connected layers, the traditional GNN module requires 280 trainable parameters, whereas the QGCN requires only 24. This compact parameterization restricts the optimization space, an effect directly reflected in the convergence trajectories under the unified training scheme. As shown in Figure 5a, the WRN + GNN architecture exhibits a diverging trend between training and validation accuracy around epoch 20. The gap between their loss curves expands from near zero in the early stages to approximately 0.14 in the later stages, with the validation loss plateauing or slightly increasing after 40 epochs while the training loss continues to decrease. This phenomenon indicates that the unconstrained model is memorizing training set noise, leading to a stagnation in its generalization capacity.

In contrast, as illustrated in Figure 5b, the training and validation curves of QG-WRN exhibit consistent generalization performance. This robustness suggests that the overfitting observed in traditional GNNs originates not only from parameter redundancy but also from the inherent limitations of their linear neighborhood aggregation mechanism. By employing modulus normalization in quantum state encoding, the QGCN introduces an intrinsic physical regularization constraint. Concurrently, it replaces traditional linear weighting with the global nonlinear unitary evolution of quantum gates. This mechanism fundamentally mitigates the impact of local noise and multi-site biases, enabling the model to extract nonlinear pathological associations within the high-dimensional Hilbert space, thereby demonstrating improved stability when addressing high-dimensional, small-sample challenges.

While evaluating these models under an identical parameter scale might intuitively seem fairer, constraining the classical GNN to merely 24 parameters is structurally impractical. Such extreme compression of hidden layer dimensions in classical networks inevitably induces a severe information bottleneck and representational collapse (underfitting), as linear mappings fundamentally rely on adequate network width to capture complex non-Euclidean topologies. Conversely, the QGCN map inputs into a 2^N^-dimensional Hilbert space using N qubits, where its parameters function not as standard linear weights but as rotation angles driving unitary evolution within an exponentially expanded state space. Therefore, the 280-parameter GNN baseline represents a necessary structural compromise to maintain minimally viable feature extraction capabilities. Ultimately, the compact parameterization of the QGCN inherently restricts the optimization space and provides robust physical regularization, directly contributing to the convergence trajectories observed during training.

#### 3.4.1. Dynamic Evolution of Trainable Quantum Phase

**Figure 6:**
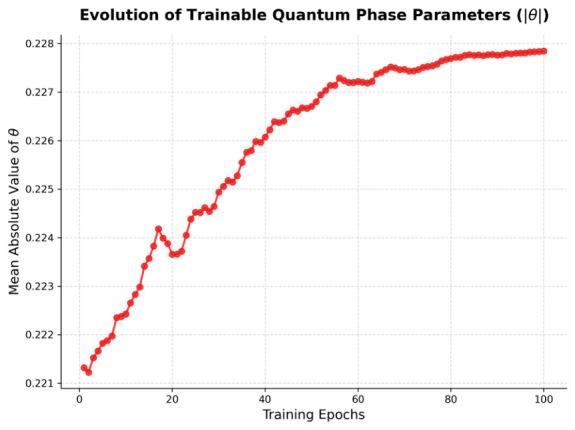
The dynamic evolution curve of trainable quantum phase parameter $\left|\theta \right|$ with training period.

In order to further verify the contribution of trainable parameters to the nonlinear expression ability of the model from the underlying physical mechanism, this study visualized the evolution trajectory of the absolute mean of the phase encoding weight θ in the VQC during the training period, as shown in Figure 6.

During the exploration phase of network initialization, the parameter $\left|\theta \right|$ oscillates to a certain extent. As the loss function continues to gradient back, the network shows an extremely significant monotonic upward trend in order to find a better classification boundary, and eventually stabilizes with the overall convergence of the model. This dynamic evolution phenomenon reveals that the QGCN module faces highly interwoven classical pathological features, and the quantum network does not perform rigid static phase mapping, but adaptively increases the scaling factor θ during training. This mechanism successfully stretches the small classical feature difference Δx exponentially into a more discriminative quantum phase difference Δϕ in the exponentially expanded Hilbert space. It is precisely this physically adaptive dynamic manifold reshaping that endows QG-WRN with the powerful ability to break through the bottleneck of classical linear aggregation.

#### 3.4.2. Structural Validation of Quantum Bit Layout

**Figure 7:**
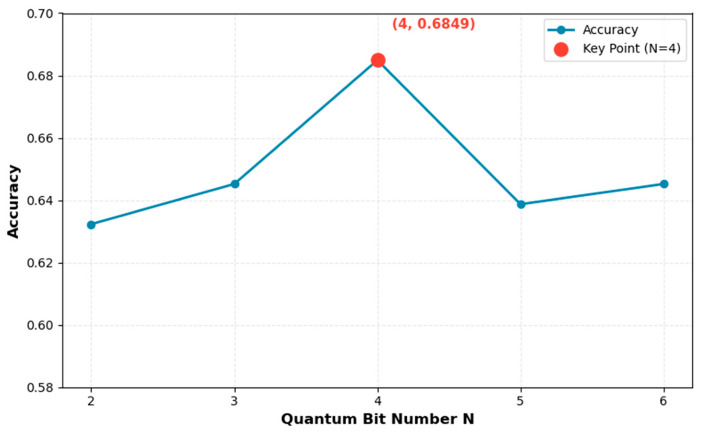
Structural validation of the QGCN module.

To investigate how the structural configuration of the variational quantum circuit fine-tunes the final classification layer boundaries, we conducted an evaluation across varying quantum bit counts (N∈{2,3,4,5,6}). As illustrated in Figure 7 and Table 4, the classification accuracy exhibits a distinct inverted U-shape trajectory in response to the expansion of the qubit layout. When the qubit count is restricted to N = 2 or 3, the model yields suboptimal accuracies. Physically, this is attributed to a severe information bottleneck; the corresponding low-dimensional Hilbert space lacks the necessary structural expressivity to decouple the highly complex, non-linear pathological connections inherent in ASD neuroimaging data. Consequently, the classification boundaries remain ambiguous and intertwined. The performance peaks significantly at N = 4, achieving an accuracy of 68.49%. At this configuration, the 2^4^ = 16 dimensional Hilbert space strikes an optimal balance. It provides sufficient parameter elasticity to adaptively stretch and reshape the topological feature manifold, thereby establishing robust and highly separable decision boundaries for the final MLP classifier. Conversely, when the layout is further expanded (N ≥ 5), a noticeable degradation in accuracy occurs. Although a larger qubit count exponentially expands the state space, it simultaneously escalates the number of trainable parameters. In the context of small-sample neuro-sensing datasets, this over-parameterization inevitably forces the network to memorize high-frequency local noise rather than generalizing true topological invariants, causing the decision boundaries to over-fit. Thus, the empirical validation firmly justifies the 4-qubit configuration as the mathematical and structural optimum for the QG-WRN architecture.

### 3.5. T-SNE Clustering

To intuitively and quantitatively evaluate the feature representation and manifold characterization capabilities of the proposed QG-WRN architecture in the deep latent space, this study combines t-SNE dimensionality reduction with three clustering evaluation metrics (Silhouette Score, Calinski-Harabasz (CH) Index, and Davies-Bouldin (DB) Index). This facilitates an in-depth 3D feature space visualization and quantitative analysis across different feature processing stages.

**Figure 8:**
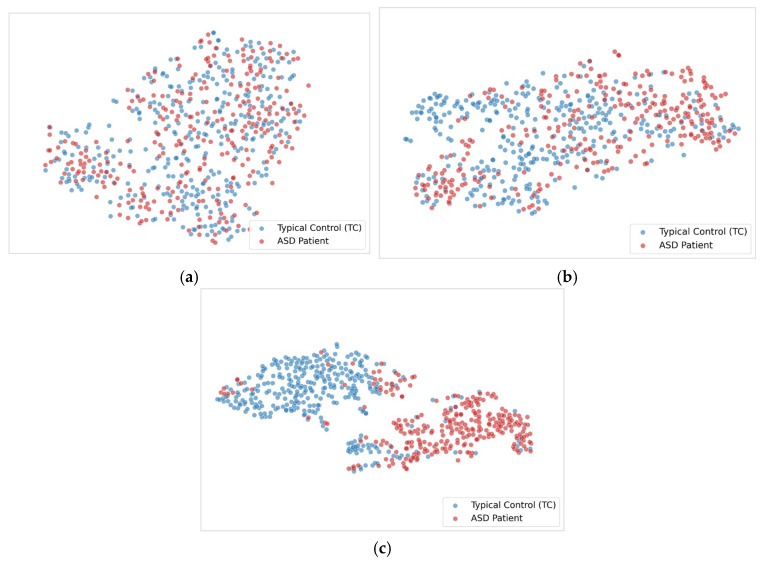
T-SNE visualization. (**a**) unprocessed raw feature; (**b**) WRN + GNN; (**c**) QG-WRN.

Observing the unprocessed raw feature space (as illustrated in Figure 8a), typical control (TC, blue dots) and ASD (red dots) subjects exhibit a highly intertwined state in the projection, yielding a Silhouette Score of only −0.001. This intertwined distribution, lacking clear geometric boundaries, reflects the inherent high-dimensional noise and substantial heterogeneity of multi-site rs-fMRI data, such as the ABIDE dataset. This complex baseline makes it difficult for shallow recognition mechanisms relying solely on raw signals to establish effective decision boundaries. Confronted with this distribution, the WRN + GNN architecture demonstrates limited feature reconstruction capability (Figure 8b). Interestingly, although this model achieved a classification accuracy exceeding 60% in macroscopic testing, its feature clustering still exhibits considerable inter-class overlap, with the Silhouette Score increasing marginally to 0.068 and the DB Index remaining relatively high at 3.654. This phenomenon reveals the underlying limitations of the linear neighborhood aggregation mechanism in traditional GNNs. During the classification phase, deep classical networks, relying on massive parameter scales, tend to fit shallow linear decision surfaces within local spaces to memorize apparent differences in the training set, thereby maintaining a basic classification accuracy. However, constrained by the low-pass filtering tendency and feature dilution effect during the linear message-passing process, the model fails to parse the hidden high-order nonlinear pathological topologies within the brain networks. Lacking an extraction of the deep geometric structure of the complex data, the extracted latent space features naturally fail to form cohesive, independent manifolds after dimensionality reduction.

In contrast, the proposed QG-WRN architecture demonstrates a substantial improvement in feature space decoupling (Figure 8c). With the introduction of the variational QGCN module operating concurrently alongside the classical wide residual network, the highly complex nonlinear pathological connections—which were previously intertwined as ambiguous neuro-sensing signals within the classical Euclidean space—are elegantly mapped into an exponentially expanded high-dimensional Hilbert space. This transformation is driven by a trainable dynamic encoding mechanism optimized during the single integrated training phase. Relying on adaptive nonlinear phase stretching and multi-qubit entanglement evolution within the quantum gate circuits, the model fundamentally overcomes the representational bottlenecks inherent to classical linear operators when processing high-dimensional neuro-sensing data. More importantly, instead of merely fitting the apparent topology, the quantum state evolution actively amplifies the inter-class distance of weak, long-range abnormal sensing features. This physically adaptive manifold reshaping ensures that the subtle neurodynamic disturbances captured by the functional imaging sensors form highly cohesive and discriminative clusters in the deep latent space.

As presented in Table 5, the Silhouette Score of the proposed method increases to 0.395. Concurrently, the CH Index, which indicates inter-cluster separation and intra-cluster compactness, improves to 490.4, while the DB Index, a measure of inter-cluster overlap, is reduced to 0.951. These highly structured dual-manifold clustering results demonstrate that the QGCN does not rely on parameter redundancy to memorize sample noise. Instead, it overcomes the nonlinear representational bottleneck, effectively extracting the core topological manifolds that drive disease classification from the highly complex medical neuro-sensing signals.

### 3.6. Model Interpretability

To investigate the physical logic and biological significance underlying the deep decision-making process, this study employs a gradient-based saliency map method [37] to interpret the QG-WRN architecture. Unlike standard 2D/3D computer vision models where class activation mapping (e.g., Grad-CAM) is typically applied, our region-based QG-WRN processes explicitly decoupled 1D neuro-sensing vectors and graphs. Therefore, this specific gradient-based approach is utilized to prevent spatial collapse and ensure precise anatomical localization. Through this retrospective feature mapping, we aim to analyze whether the nonlinear mathematical boundaries captured by the model align with existing neuropathological priors.

**Figure 9:**
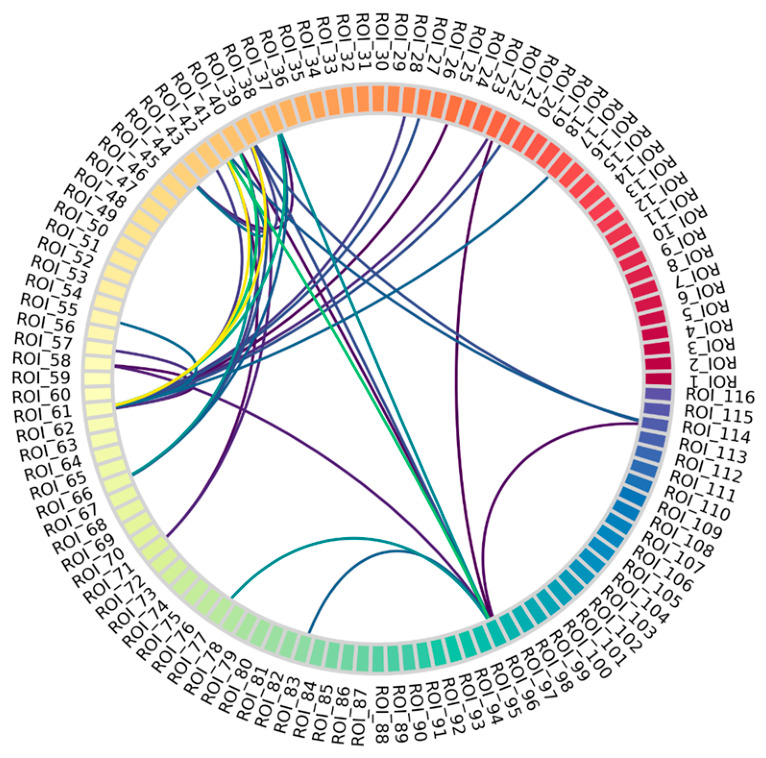
All 34 selected functional connection features shown in the circos plot.

Figure 9 details the top 34 salient functional connections contributing most significantly to the ASD diagnostic classification. In traditional graph learning paradigms, due to the inherent low-pass filtering tendency of linear neighborhood aggregation, high-magnitude local short-range connections often obscure weak long-range topological signals (i.e., the feature dilution effect). However, the edge distribution in the chord diagram indicates that the key features selected by the model are not confined to adjacent local brain regions; instead, they are widely distributed across heterogeneous networks spanning considerable anatomical distances. This phenomenon suggests that the variational QGCN module effectively preserves and amplifies these weak, nonlinear long-range associations—which are frequently filtered out by traditional operators—via the adaptive phase stretching mechanism within the high-dimensional Hilbert space.

**Figure 10:**
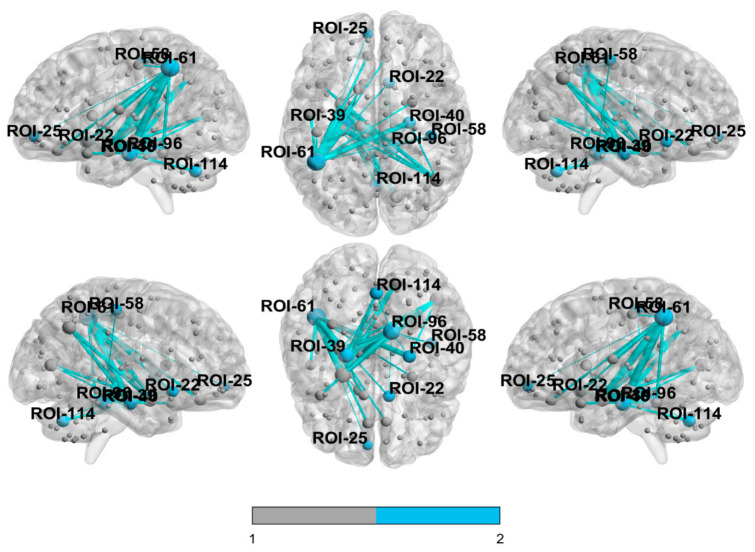
Visualization of prominent network centrality and discriminative hubs within the deep latent space.

Figure 10 visualizes the core discriminative hubs constituting these abnormal circuits. Mapped onto standard AAL116 atlas coordinates, specific anatomical regions (represented by larger spherical nodes) exhibit prominent network centrality within the deep latent space. Specifically, the model identifies regions responsible for advanced social cognition and language processing, such as the left inferior temporal gyrus (ROI-61), left angular gyrus (ROI-39), and bilateral superior temporal gyri (ROI-21/22), as core nodes of large-scale network topological imbalance. This abnormal connection pattern across the temporoparietal cortex avoids the computational pitfall of feature smoothing and aligns consistently with current clinical observations in cognitive neuroscience regarding impaired connectivity in the default mode network (DMN) of ASD patients [38].

Notably, the model assigns prominent saliency weights to the right cerebellar Crus II (ROI-96) and cerebellar lobule X (ROI-114). Such atypical high-order mappings extending from the cerebellum to high-level cortical regions frequently elude classical networks that rely on shallow feature fitting. Modern fMRI research increasingly recognizes that cerebellar function transcends motor control; its structural dysfunction within social and emotional circuits is now considered a critical lead in deciphering the pathological heterogeneity of ASD [39]. Quantitative distributions in Table 6 indicate that high-weight discriminative features transcend the linear stacking of adjacent nodes, exhibiting significant cross-network interaction properties. Specifically, ROI-39 within the DMN forms prominent connections with the cerebellar ROI-96 and ROI-114. This distribution suggests that QG-WRN identifies not only local imbalances within sensory and cognitive networks but also the long-range synergistic failure between cerebellar circuits and large-scale cognitive networks (e.g., the DMN).

These findings align with the emerging consensus regarding cerebellum-cortex dysconnectivity in ASD [40,41] and provide computational evidence for the value of the dual-stream quantum architecture. By leveraging the Hilbert space to structurally amplify weak cross-network pathological priors—which are often obscured by “feature dilution” in classical GNNs—the QG-WRN effectively establishes robust decision boundaries for automated ASD diagnosis.

## 4. Discussion

The empirical success of the QG-WRN architecture in ASD diagnosis invites a deeper reflection on the role of quantum computing in neuroimaging. From a theoretical perspective, the effectiveness of the QGCN module in capturing pathological topological failures may be attributed to the dimensional elasticity of the Hilbert space. While classical GNNs represent brain network features in a Euclidean space ${\mathbb{R}}^{d}$ and aggregate information via linear message-passing, our quantum approach maps these features into a 2^N^-dimensional Hilbert space. In this exponentially expansive space, the non-linear evolution driven by quantum entanglement gates allows the model to construct a more discriminative feature manifold for sparse and long-range pathological connections—signals that are often smoothed out by classical linear operators.

While the current globally pooled evaluation effectively validates the fundamental synergy of the quantum-classical architecture, we acknowledge that it does not explicitly decouple specific inter-site scanner variances. Future work will incorporate Leave-One-Site-Out (LOSO) cross-validation to further optimize and evaluate the model’s domain-transferability across independent clinical sites.

For real quantum hardware deployment, an operational validation protocol should include calibration-aware circuit transpilation, repeated measurement under multiple shot budgets, readout-error mitigation, noise-aware parameter initialization, and drift monitoring. If measured output distributions deviate beyond a predefined tolerance from noiseless simulation, recalibration or fallback to the classical branch should be triggered. Therefore, deploying and rigorously evaluating the QG-WRN architecture on real physical quantum backends constitutes a primary focus of our future work. Transitioning from high-fidelity classical simulations to physical deployments will inherently require addressing hardware-specific noise fluctuations. Investigating the impact of actual hardware noise topologies on diagnostic robustness will be a crucial next step. To ensure operational reliability in future clinical applications, it will be necessary to integrate standard Quantum Error Mitigation (QEM) protocols—such as Zero-Noise Extrapolation (ZNE) to estimate noise-free expectation values, and Readout Error Mitigation (REM) to correct measurement biases. Implementing these noise compensation strategies alongside our inherently shallow circuit design (L = 2) will form the basis of a comprehensive validation protocol, ultimately bridging the gap between theoretical quantum advantage and clinical applicability in neuroimaging.

The proposed QG-WRN architecture is not strictly limited to fMRI. The fundamental prerequisite for our model is that the raw medical data can be abstracted into a graph structure comprising node-level features (processed by the WRN) and edge-level topological connections (processed by the QGCN). Therefore, this algorithm can be seamlessly extended to other neuro-sensing modalities that naturally output multi-channel time-series data, such as Electroencephalography (EEG) and Magnetoencephalography (MEG). Extension to structural MRI, diffusion tensor imaging, or multimodal imaging would require modality-specific graph definitions, harmonized feature normalization, and a revised fusion layer. In particular, DTI would introduce structural connectivity edges rather than functional correlation edges.

However, we regard this study primarily as a preliminary exploration of the potential advantages offered by quantum methods in this domain. While our results suggest that quantum-enhanced kernels can provide higher parameter efficiency and better generalization in small-sample scenarios, the precise mathematical origins of this quantum advantage and its generalizability across different neuroimaging modalities still warrant extensive investigation. This work provides a starting point, but the “black-box” nature of certain quantum-classical interactions means that the interpretability of quantum feature mappings is an area that requires more rigorous theoretical refinement.

Additionally, it is crucial to acknowledge the impact of brain atlas selection on graph configuration stability. In this study, we utilized the strictly anatomically defined AAL116 atlas. Recent investigations in functional neuroimaging indicate that while denser atlases (e.g., CC200 or Schaefer) provide finer functional granularity, coarser macro-anatomical atlases like AAL are significantly less prone to overfitting and maintain stable spatial margins across heterogeneous multi-site datasets. For our hybrid architecture, AAL116 offers an optimal equilibrium: it preserves essential neuropathological topologies without overwhelming the compact 4-qubit VQC. Processing highly dense functional parcellations would require deeper quantum circuits, inevitably introducing severe gate noise and decoherence given current Noisy Intermediate-Scale Quantum (NISQ) constraints. Nevertheless, as fault-tolerant quantum hardware matures, investigating how alternative or multi-scale atlas configurations influence the quantum feature manifold remains a highly promising trajectory for future research.

Furthermore, the architectural configuration of our variational quantum circuit is deliberately designed to align with the stringent hardware constraints of the current NISQ era. Unlike deep quantum neural networks that demand fault-tolerant hardware, our QGCN module employs a highly compact 4-qubit architecture with merely L = 2 evolution layers, comprising exactly 24 single-qubit rotation gates and 8 two-qubit CZ gates. This structurally lean design minimizes both memory and computational footprints, strictly restricting the accumulation of quantum gate errors and decoherence effects. By prioritizing high parameter efficiency within a restricted optimization space, the model demonstrates strong theoretical feasibility for near-term quantum devices. While deploying this hybrid model onto real physical edge platforms or quantum backends entails hardware-specific transpilation and quantum error mitigation (QEM) that fall beyond the current scope, its theoretical footprint strongly supports its future physical deployability. Ultimately, this compact configuration establishes a highly resource-efficient algorithmic foundation, demonstrating the theoretical feasibility and potential readiness of the QG-WRN architecture for future diagnostic integration within advanced neuro-sensing edge systems once physical hardware and low-level control protocols mature.

Despite these structural optimizations, we acknowledge that the current empirical validations rely on high-fidelity classical simulations. While this provides a controlled environment to verify the mathematical limits of quantum state evolution in parsing high-order brain topologies, the transition to physical hardware introduces complex noise dynamics. Therefore, deploying and rigorously evaluating the QG-WRN architecture on real physical quantum backends constitutes a primary focus of our future work. Investigating the impact of actual hardware noise topologies on the diagnostic robustness will be a crucial next step in bridging the gap between theoretical quantum advantage and clinical applicability in neuroimaging.

## 5. Conclusions

To address the representational limitations and generalization challenges when confronting the dual heterogeneity of ASD brain networks, this study proposes a cross-paradigm dual-stream synergistic diagnostic model. The results demonstrate that by decoupling ALFF and FC, and introducing a variational quantum circuit to process complex network topologies, the proposed architecture facilitates the characterization of deep pathological features with significantly higher parameter efficiency. This approach exhibits competitive performance across multiple evaluation metrics, and its decision-making logic aligns with established neurobiological priors. Overall, this hybrid architecture represents an effective refinement to classical computer-aided diagnosis systems. Beyond providing a practical tool for ASD identification, this work serves as an exploratory step toward realizing quantum advantage in medical imaging, highlighting a viable pathway for addressing the conflict between computational complexity and small-sample data constraints.

Accordingly, the current framework should be interpreted as an algorithm-centric, rs-fMRI-focused hybrid diagnostic model with bounded scalability. While the retrospective evaluation on the ABIDE I cohort thoroughly validates its theoretical and architectural efficacy, transitioning this proof-of-concept into real-world clinical practice requires critical intermediate steps to address out-of-distribution variability. Future endeavors must encompass rigorous validation on independent external clinical cohorts to verify cross-site generalizability, alongside prospective clinical trials. Furthermore, its extension to multi-modal neuroimaging and physical quantum hardware remains feasible but necessitates comprehensive empirical validation of graph construction, qubit layout, noise compensation, and modality-specific fusion protocols prior to actual clinical deployment.

## Figures and Tables

**Table 1:** Comparison of classification performance of SOTA methods. The use of bold text in the table highlights the optimal experimental results and the method proposed in this article.

Methods	ACC (%)	SEN (%)	SPE (%)
SVM	62.58 ± 1.24	60.34 ± 1.51	71.76 ± 1.44
Random Forest	58.32 ± 2.06	55.18 ± 1.67	61.49 ± 3.01
MLP	60.13 ± 1.38	56.19 ± 1.02	65.18 ± 8.66
1D-CNN	56.17 ± 2.87	55.14 ± 1.15	57.14 ± 1.41
WRQDCNN [31]	61.62 ± 1.92	59.57 ± 5.93	64.91 ± 4.24
DRBMs [32]	64.43 ± 1.93	56.86 ± 2.10	71.93 ± 3.54
Video MAE [33]	64.04 ± 1.50	54.04 ± 0.76	68.10 ± 0.65
SSAE [34]	64.15 ± 1.41	63.27 ± 1.38	67.65 ± 1.58
BrainGNN [35]	60.65 ± 2.72	60.91 ± 1.87	57.37 ± 1.23
AL-NEGAT [36]	64.25 ± 4.24	**66.25 ± 4.36**	61.71 ± 4.80
**proposed**	**68.49 ± 1.34**	63.26 ± 1.24	**74.29 ± 2.04**

**Table 2:** Ablation experiment results. The use of bold text in the table highlights the optimal experimental results and the method proposed in this article.

Methods	ACC (%)	SEN (%)	SPE (%)	F1 (%)
w/o QGCN	52.90 ± 1.47	54.11 ± 5.22	58.68 ± 7.68	59.63 ± 3.66
w/o WRN	61.42 ± 1.80	59.17 ± 6.82	62.64 ± 4.50	57.84 ± 4.17
w/o Reordering	62.72 ± 1.65	58.56 ± 1.97	66.84 ± 2.10	55.88 ± 3.74
w/o Trainable Parameters	64.46 ± 2.56	60.49 ± 3.82	68.33 ± 3.28	50.37 ± 5.98
w/o Quantum Layer	61.16 ± 3.68	52.99 ± 1.16	68.73 ± 3.75	62.22 ± 0.00
**proposed**	**68.49 ± 1.34**	**63.26 ± 1.24**	**74.29 ± 2.04**	**62.22 ± 1.57**

**Table 3:** Parameter scale of GNN and QGCN.

Methods	Total Number of Parameters (Excluding Fully Connected Layers)
GNN	280
QGCN	24

**Table 4:** Experimental results of Structural validation of the QGCN module. Bold values indicate the best performance.

Quantum Bit Number (N)	ACC (%)	SEN (%)	SPE (%)	Total Number of Quantum Parameters
2	63.23	60.00	67.00	12
3	64.52	**65.71**	64.49	18
5	63.87	61.43	66.78	30
6	64.52	58.57	70.34	36
**4**	**68.49**	63.26	**74.** **29**	**24**

**Table 5:** T-SNE clustering metrics. The use of bold text in the table highlights the optimal experimental results and the method proposed in this article.

Methods	Silhouette Score	CH	DB
no processed	−0.001	0.1	73.261
WRN + GNN	0.068	38.5	3.654
**proposed**	**0.395**	**490.4**	**0.951**

**Table 6:** Connections among the top 10 brain regions.

ROI Pair	Anatomical Name	Associated Functional Network	Connection Weight
ROI 21—ROI 61	Superior Temporal Gyrus (L)—Inferior Temporal Gyrus (L)	Auditory/DMN—DMN/Visual	0.001578
ROI 39—ROI 114	Angular Gyrus (L)—Cerebellum 10 (R)	DMN—Cerebellar	0.001576
ROI 39—ROI 96	Angular Gyrus (L)—Cerebellum Crus II (R)	DMN—Cerebellar	0.001575
ROI 28—ROI 61	Rectus Gyrus (R)—Inferior Temporal Gyrus (L)	Limbic/DMN—DMN/Visual	0.001542
ROI 22—ROI 61	Superior Temporal Gyrus (R)—Inferior Temporal Gyrus (L)	Auditory/DMN—DMN/Visual	0.001518
ROI 58—ROI 96	Postcentral Gyrus (R)—Cerebellum Crus II (R)	Somatomotor—Cerebellar	0.001498
ROI 58—ROI 61	Postcentral Gyrus (R)—Inferior Temporal Gyrus (L)	Somatomotor—DMN/Visual	0.001472
ROI 96—ROI 114	Cerebellum Crus II (R)—Cerebellum 10 (R)	Cerebellar—Cerebellar	0.001471
ROI 40—ROI 96	Angular Gyrus (R)—Cerebellum Crus II (R)	DMN—Cerebellar	0.001467
ROI 25—ROI 61	Orbital Medial Frontal Gyrus (L)—Inferior Temporal Gyrus (L)	Limbic/DMN—DMN/Visual	0.001461

## Data Availability

In this research, a public dataset was used, which can be found at: https://fcon_1000.projects.nitrc.org/indi/abide/, accessed on 29 December 2025.
